# Navigating prognostic strategies for GH- and PRL-secreting pituitary neuroendocrine tumors: key insights from a clinicopathological study

**DOI:** 10.3389/fendo.2025.1541514

**Published:** 2025-04-10

**Authors:** Roxana-Ioana Dumitriu-Stan, Iulia-Florentina Burcea, Ramona Dobre, Valeria Nicoleta Nastase, Raluca Amalia Ceausu, Catalina Gabriela Molnar, Marius Raica, Catalina Poiana

**Affiliations:** ^1^ Department of Endocrinology, ‘Carol Davila’ University of Medicine and Pharmacy, Bucharest, Romania; ^2^ Deparment of Endocrinology I, ‘C. I. Parhon National Institute of Endocrinology, Bucharest, Romania; ^3^ Department of Microscopic Morphology/Histology, ‘Victor Babes’ University of Medicine and Pharmacy, Timisoara, Romania; ^4^ Angiogenesis Research Centre, ‘Victor Babes’ University of Medicine and Pharmacy, Timisoara, Romania; ^5^ Deparment of Internal Medicine, Florida Atlantic University, Boca Raton, FL, United States

**Keywords:** pituitary tumor, 2022 WHO classification, acromegaly, prolactinoma, prognostic factors

## Abstract

**Background:**

The classification of pituitary neuroendocrine tumors (PitNETs), also known as pituitary adenomas, has progressed significantly since 2004. The PitNET lineage now serves as the foundation of the classification. We investigated the prognostic value of clinicopathological markers in a cohort of patients diagnosed with acromegaly and prolactinomas who underwent transsphenoidal tumor resection.

**Methods:**

A total of 50 patients (45 patients with confirmed acromegaly and 5 with prolactinomas) in evidence at ‘C. I. Parhon National Institute of Endocrinology (Pituitary and Neuroendocrine Pathology Department, Bucharest, Romania), who underwent tumor resection between 2010 and 2023, was recruited, with a median follow-up time of 7.02 years (IQR: 3–10). Surgical samples were stained for anterior pituitary hormones, ki-67 labeling index, CAM 5.2 expression, and the following transcription factors (TFs): steroidogenic factor (SF-1), T-box family member TBX19 (TPIT) and POU class 1 homeobox 1 (PIT-1). Additionally, somatostatin receptor 5 (SSTR 5) and 2 (SSTR 2) expression was evaluated in all patients.

**Results:**

Based on the 2022 WHO classification, the majority of cases were PIT-1 lineage tumors (n=40, 72.7%), followed by TPIT-lineage (n=4, 7.3%), and SF-1 lineage (n=3, 5.5%) and 14.5% (n=4) were classified as tumors with no distinct cell lineage (NDCL). In the multivariate Cox regression analysis, the postoperative GH value was independently associated with the outcome (HR 1.042, 95% CI 1.004–1.081, p=0.030), as well as the postoperative PRL value (HR 1.95% CI 1,1.001, p=0.019), the ki-67 labelling index (HR 2.43, 95% CI 1.109–5.330, p=0.026). Other factors associated as well with the success of the treatment were the postoperative tumor diameter (HR 1.038 95% CI 0.997–1.080, p=0.068) and the expression of SSTRs 2 and 5. Combining the four parameters, ki-67, SSTR 2, SSTR 5, GH, IGF-1 and the maximal tumor diameter (postoperative values), we established a prediction model with an AUC of 0.924 and relatively high sensitivity and specificity.

**Conclusion:**

A clear classification system that can guide clinical and neurosurgical management of patients with GH- and PRL-secreting PitNETs is not currently available, but certain clinicopathological factors can be used to predict patient prognosis. In our study, somatostatin receptor expression, ki-67, and postoperative values of GH and IGF-1, as well as the maximal postoperative tumor diameter, were the strongest predictors of outcome.

## 1 Introduction

The classification of pituitary neuroendocrine tumors (PitNETs, also known as pituitary adenomas) has progressed significantly since 2004. The evolution of classification systems has culminated with the use of transcription factors (TFs) immunohistochemistry (IHC), and the latest WHO classification for 2022 recommends a cell lineage-based system (1). Scientists have proposed several algorithms that can aid in the diagnosis and management of these tumors. The algorithms are based on IHC for TF and anterior pituitary hormones or IHC for certain hormones on the basis of clinical features and hormonal excess, with the evaluation of TF only for unclear cases and the third approach involving screenings composed of TFs with lineage-specific hormone IHC testing (2–5).

A group of researchers composed of anatomopathologists, endocrinologists and researchers involved in the pathology of sellar formations was established in 2016 by the European Pituitary Pathology Group (EPPG). For a more accurate diagnosis, the EPPG recommended a multistep approach that includes clinical and neuroimaging elements, IHC for TF and proliferation markers and, when necessary, the use of predictive markers for treatment response. Multidisciplinary team management involving endocrinologists, radiologists, neurosurgeons, geneticists and oncologists is recommended (6). The first step in the proposed assessment is the evaluation of the clinical characteristics of the patients, followed by histopathological examination (H&E examination) and an entire panel of IHC for pituitary hormones followed by IHC for low-molecular-weight cytokeratin. The algorithm relies on hormones alone to detect somatotroph, mixed GH/PRL, lactotroph, thyrotroph, corticotroph, and gonadotroph types. The third step included the evaluation of mitotic activity, the MIB/ki-67 labeling index, and the last step for the evaluation of tumors negative for pituitary hormones. Negative tumors for the three TFs were assigned to null cell tumors. The aggressive PitNETs were defined by the group on the basis of a clinicopathological system, and IHC was suggested for markers that may guide treatment, such as somatostatin receptors. The EPPG algorithms are limited by the renewed focus on TF assessment in defining the PitNET lineage in the WHO 2022 classification and the risk of missing rarer tumors with “no distinct cell lineage” that may express TFs but stain only for one hormone (7). Additionally, the EPPG proposed the term somatotroph plurihormonal PIT1-positive tumors to define lesions with acromegaly/gigantism that show variable expression of PIT1, GH, TSH and/or PRL and thyrotroph plurihormonal PIT1-positive tumors to define those presenting with central hyperthyroidism. The clinical management of these tumors is different.

The five-tiered classification proposed in 2013 by a group of French researchers was validated in numerous studies with a total number of patients of 2206 (8). This classification is based on the tumor diameter, the type of tumor and the grade of the tumor. The 5 grades are as follows: grade 1a: noninvasive and nonprovasive tumors; grade 1b: noninvasive and proliferative tumors; grade 2a: invasive and nonproliferative tumors; grade 2b: invasive and proliferative tumors; and grade 3: metastatic tumors.

Few studies have evaluated which factors can accurately predict patient outcomes. Age, sex, preoperative GH and IGF-1 levels, maximal tumor diameter, Hardy’s and Knosp’s classifications, MRI T2-weighted tumor intensity, and cytokeratin expression pattern, as well as the experience of the neurosurgeon, are frequently related to postoperative outcomes. Younger age, higher preoperative GH and/or IGF-1 levels, group 2b clinicopathological classification, Knosp grade IV, MRI, T2-weighted tumor hyperintensity and sparsely granulated cytokeratin expression patterns are also related to worse postoperative outcomes (9–11). Somatostatin receptor expression (SSTR2 and 5) is also an important factor in the treatment response of patients diagnosed with GH-secreting PitNETs. GH-secreting pituitary adenomas express different subtypes of SSTRs, predominantly SSTRs 2 and 5, which play major roles in reducing GH secretion and insulin-like growth factor I (IGF-I) levels (12, 13). The expression of SSTR2 is correlated with the efficacy of somatostatin analogs (SSAs) in suppressing GH and IGF-I levels *in vitro* and with IGF-I normalization in acromegalic patients (14).

To date, few studies have developed accurate models that can fit patients’ needs and predict disease evolution. The recognition of the heterogeneous and sometimes unpredictable evolution of these tumors is a step forward and once again accounts for the shift in the definition and terminology for pituitary adenomas.

The aim of our study was to identify which factors correlate with patient outcomes and to identify a prediction model that can be applied in these cases.

## 2 Materials and methods

### 2.1 Patients

We conducted a retrospective, observational study following the Declaration of Helsinki and approved by the Institutional Ethics Committee of ‘C. I. Parhon’s National Institute of Endocrinology, Bucharest, Romania (Ethics Approval no. 04/24.02.2022). We included 50 patients with a confirmed diagnosis of acromegaly or prolactinoma (PRL- and mixed PRL- and GH-secreting PitNETs) with evidence at ‘C. I. Parhon’ National Institute of Endocrinology (Pituitary and Neuroendocrine Pathology Department, Bucharest, Romania), who underwent pituitary neurosurgical intervention at the Neurosurgery Clinic of ‘Bagdasar Arseni’ Emergency Clinical Hospital (Bucharest, Romania), the Neurosurgery Clinic of ‘Colentina’ Hospital (Bucharest, Romania), the Neurosurgery Clinic of Brain Institute, Monza Hospital (Bucharest, Romania), or the NeuroHope Clinic (Bucharest, Romania). The patients underwent transsphenoidal interventions between 2010 and 2023. The inclusion criteria were as follows: age > 18 years, a diagnosis of acromegaly or prolactinoma who underwent transsphenoidal intervention and had available tumor paraffin blocks and patients with follow-up for at least one year. The exclusion criteria were patients diagnosed with acromegaly or prolactinoma who did not undergo neurosurgical resection; patients who did not have tumor paraffin blocks available or no viable pituitary tissue or insufficient tissue for staining; and patients with known germline mutations who were carriers of hereditary syndromes.

### 2.2 Variables

The clinical data were retrospectively included (date of diagnosis, date of neurosurgical intervention, symptoms, comorbidities, such as diabetes mellitus, arterial hypertension, dyslipidemia, type of treatment, preoperative treatment, body mass index, date of control of disease, surgical complications), and hypopituitarism. Paraclinical data included the nadir GH in the oral glucose tolerance test (OGTT), random GH, the IGF-1 index (the ratio of the measured value of IGF-1 to the upper normal limit for age and sex), the maximal tumor diameter at diagnosis and postoperative or pre- and postradiotherapy, and PRL levels. The Knosp grade was established on the basis of preoperative MRI/CT, as previously described in the literature (9). Invasive tumors were defined as those with cavernous or sphenoidal sinus invasion or with more than 50% carotid encasement by the tumor. A giant tumor was defined as a tumor with any dimension greater than 4 cm. Gross total resection (GTR) was defined as the absence of residual tumor on the first postoperative imaging evaluation.

GH levels were measured via a chemiluminescence assay (Liason, Sallugia, Italy) with a sensitivity of 0.05 ng/ml. Serum IGF-1 was measured via a Liaison IGF-1 chemiluminescence assay (DiaSorin, Sallugia, Italy) with a sensitivity of 15 ng/ml.

The PRL level was measured on a Cobas E602 immunoanalyzer via the Elecsys Prolactin II Kit of Roche Diagnostics, Mannheim, Germany. The measurement range was 0.047–470 ng/mL (1.00–10000 mIU/L). No high-dose hook effect was found up to 12690 ng/mL (270000 mIU/L).

### 2.3 Diagnosis and inclusion criteria

The diagnosis of acromegaly was established on the basis of the Endocrine Society clinical practice guidelines and the national protocol for the diagnosis and therapeutic management of acromegaly which were available at the time of diagnosis: clinical features of GH excess and failure of GH suppression below 0.4 ng/ml in the 75 g OGTT and elevated IGF-1 for age and sex for the patients which were diagnosed strating with 2021 when the ultrasensitive assays were available in our clinic (n=5). Disease control was defined as random GH <1 ng/ml or nadir GH in the OGTT  < 0.4 ng/ml and IGF-1 < ULN (upper normal limit) for age and sex, at the follow-ups after 2021 (10–14). For the patients diagnosed until 2020 the diagnosis was based using the following criteria: IGF-I elevated for age and sex, random GH > 2.5 ng/ml and failure of GH suppression below 1 ng/ml in the 75 g OGTT (n=40).

Patients with discrepant GH and IGF-1 concentrations at diagnosis were excluded from the study. Postoperative, discrepant GH and IGF-1 concentrations were also present in few cases and were considered partially controlled under medical treatment (n=5) (13). Patients who underwent treatment with pegvisomant were evaluated using only IGF-1 (n=5).

The diagnosis of prolactinoma was established on the basis of the Pituitary Society guidelines for the diagnosis and management of PRL-secreting adenomas in patients with clinical signs and symptoms of hyperprolactinemia (oligomenorrhea or amenorrhea with or without galactorrhoea in women and erectile dysfunction in men, loss of libido and infertility in both sexes) (11). PRL was measured by immunoassay, calibrated against the WHO 84/500 international standard containing exclusively 23 kDa monomeric human prolactin with sex-specific reference intervals. Patients with other causes of hyperprolactinemia were excluded.

Resection of the prolactinoma by an expert pituitary surgeon was recommended in the following cases: patients who do not exhibit rapid improvement in neuro-ophthalmologic impairment after two weeks of cabergoline treatment, patients who are resistant/intolerant to cabergoline or other dopamine agonists (DAs), patients who escape from DA effects, or patients who require treatment but are unwilling to receive chronic medical therapy (12, 13). DA resistance is defined as the failure to normalize PRL levels and achieve at least a 50% tumor size reduction at the maximally tolerated doses of DA. The suggested maximum dose of cabergoline is approximately 4 mg per week. At least 6 months of the highest tolerated DA dose is suggested as the minimum duration of treatment. Partial resistance to DA is defined as a decrease in the tumor size and prolactin levels without normalization, requiring a higher dose of DA to achieve a complete response. Complete resistance to DA is defined as failure to obtain normal prolactin values, a failure to reduce tumor size by 50%, and/or failure to regain fertility with maximum tolerated doses of DA (12).

Surgery is also recommended as initial therapy for patients with pituitary apoplexy with severe clinical symptoms, acute intracranial hypertension, or massive extrasellar extended adenomas with a high risk of visual impairment. For refractory cases, radiotherapy, especially gamma knife radiosurgery, and temozolomide treatment are also considered.

In the cases included in our study (n=5 prolactinomas) surgery was considered as following: pituitary apoplexy – 1 case, absence of biochemical control under medical treatment with cabergoline – 4 cases.

Pituitary hormone deficiency was defined as secondary hypothyroidism (low free T4 and low/normal TSH), hypogonadotropic hypogonadism with low estradiol (in women) or low testosterone (in men) with low/normal FSH and LH), and secondary adrenal failure was defined as a serum 8 A. M. cortisol level less than 5 µg/dl or a positive test in a 1 microgram short Synachten test.

Patients were considered cured if they did not have residual or recurrent tumors after neurosurgical intervention throughout the observation period. Recurrence was considered when the tumor/hormonal hypersecretion reappeared after the patient was cured.

The short-term outcomes, which were determined approximately 3 months postoperatively, were as follows: biochemical remission in the absence of adjuvant medical treatment and evaluation of the residual tumor via magnetic resonance imaging (MRI) or computed tomography (CT). The patients had an average follow-up period of 7.02 years (IQR 3–10).

The patients who did not achieve biochemical control after surgery received only one of the following secondary treatments: repeated surgery, radiotherapy, medical or combined therapy. The criteria for postoperative biochemical remission were based on the established guidelines: random serum GH <1 ng/ml, glucose inhibition test with a nadir GH <0.4 ng/ml and normalized serum IGF-1 for age and sex for acromegaly patients. At follow-up, patients with discrepant GH and IGF-1 levels were considered to have a partial control under medical treatment (n=10).

The criterion for postoperative biochemical remission for prolactinoma patients was normalization of prolactin (< 1.0xULN). Clinical remission was defined as the restoration of gonadal function and the resolution of complaints (with no additional treatment needed) without normalization of prolactin. Patients who were not in biochemical or clinical remission were perceived to have persistent disease.

We considered the possible predictors for remission and relapse on the basis of previous studies, biological plausibility, and availability of data: age at diagnosis and surgery; sex; serum (nadir) GH, IGF-1, and PRL concentrations at diagnosis; tumor size (micro- or macroadenoma at diagnosis); cavernous sinus invasion (Knosp grade); preoperative medical treatment; and IGF-1, GH, and PRL levels measured postoperatively, values that were measured at least 12 weeks after surgery; and postoperative tumor diameter. Additionally, we included IHC factors that could predict patient outcome.

### 2.4 Histopathological and IHC analysis

Using postoperative tumor paraffin blocks, we performed morphological and immunohistochemical analyses. In **Table 1**, we present the antibodies used for the immunohistochemical analysis. Bond Epitope Retrieval Solutions 1 and 2, with pH values of 6 and 9, were used for unmasking (Leica Biosystems, Newcastle Ltd., Newcastle Upon Tyne NE 12 8EW, UK), and 3% hydrogen peroxide was used to block endogenous peroxidase for 5 min.

**Table 1:** Antibodies used for IHC analysis.

Antibody	Company	Clone	Dilution Factor	Expression Pattern
GH	Dako, Agilent	Polyclonal rabbit anti-human	1:400	Cytoplasmatic
PRL	Dako, Agilent	Polyclonal rabbit anti-human	1:300	Cytoplasmatic
ACTH	Dako, Agilent	C93	1:50	Cytoplasmatic
FSH	Thermo Fisher Scientific	FSH03	1:500	Cytoplasmatic
LH	Thermo Fisher Scientific	LH01	1:500	Cytoplasmatic
TSH	Thermo Fisher Scientific	TSH01 + TSH02	1:400	Cytoplasmatic
Ki-67	Thermo Fisher Scientific	MM1, RTU	-	Nuclear
Cytokeratin Cam 5.2	Diagnostic BioSystems	CAM5.2, RTU	-	Cytoplasmatic
Pit-1	Thermo Fisher Scientific	Rabbit polyclonal antibody	1:1500	Nuclear
TPIT	Abcam	Mouse monoclonal	1:1000	Nuclear
SF 1	Abcam	Rabbit recombinant monoclonal	1:1000	Cytoplasmatic
SSTR2	Abcam	Rabbit recombinant monoclonal	1:1000	Membrane
SSTR5	Abcam	Rabbit recombinant monoclonal	1:1000	Membrane

GH, growth hormone; PRL, prolactin; ACTH, adrenocorticotropic hormone; TSH, thyroid-stimulating hormone; FSH, follicle-stimulating hormone; LH, luteinizing hormone; Pit-1, pituitary-specific transcription factor Pit-1; Dako, Agilent, Santa Clara, CA, USA; Thermo Fisher Scientific, Waltham, MA, USA; Diagnostic Biosystems, Pleasanton, CA, USA; Abcam, Waltham, MA, USA.

All the tissue samples were within the limits of the standard dimensions and were less than 1 cm^3^. The sampling was followed by all the steps of the primary processing, thus obtaining the paraffin blocks. The histopathological diagnosis was established after routine hematoxylin and eosin (H&E) staining of 3 µm sections from each sample. Microscopic examination was performed with a Nikon Eclipse E 600 microscope (Nikon Corporation, Tokyo, Japan).

Immunohistochemical (IHC) reactions were assessed at the cellular level. The immunohistochemical expression of GH, PRL, TSH, ACTH, FSH, and LH was analyzed at the cytoplasmic level, along with the expression of Ki-67, PIT-1, TPIT and SF-1 in the nucleus. Stains for the 6 pituitary hormones were scored in a blinded fashion. The proportion score for anterior pituitary hormones was quantified according to the following criteria: score 0 (0–10% positive cells), score 1+ (10–30% positive cells), score 2+ (30–60% positive cells), and score 3+ (>60% positive cells). The intensity scores used ranged from 0 to 3+ (from absent to strongly stained). Staining greater than 10% was considered positive for the purpose of interpreting the results.

The staining of the transcription factors (TFs) PIT-1, TPIT, and SF-1 was quantified by three observers under a multihead microscope, and the results are expressed as the percentage and intensity of PIT-1-positive cells. The scoring criteria used were as follows: 0 (0–10%), 1+ (10–30%), 2+ (30–60%), 3+ (60–80%), and 4+ (80–100%). Staining of more than 10% of the tumor cells was considered positive, excluding marginal areas adjacent to the adenohypophysis.

The number of cells positive for Ki-67 in the nucleus was quantified via optical microscopy (magnification ×20) via ImageJ version 2.0 (a semiautomatic evaluation that excluded the nuclei of endothelial and stromal cells). Additionally, the CAM 5.2 expression pattern and somatostatin receptor 2 and 5 expression were evaluated.

Immunostaining with SSTR 2 and SSTR 5 was reported in a semiquantitative manner via an immunoreactivity scoring system, and only membranous staining was recorded (13, 14). The immunoreactivity score (IRS) was determined by calculating the product between the percentage of positive tumor cells (0 = none; 1 < 10%; 2 = 10–50%; 3 = 51–80%; 4 > 80%) and the staining intensity (0 = none; 1 = weak; 2 = moderate; 3 = strong) to obtain a score between 0 and 12 (0–3 = low score; 4–6 = intermediate score; 7–12 = high score).

The pattern of staining with CAM5.2 was reported and determined according to the following criteria: perinuclear or ring-like pattern (absent, < 70%, or ≥ 70%), dot pattern/fibrous bodies (absent, 1–8%, 9–69%, or ≥ 70%), and transitional pattern (absent, < 70%, or ≥ 70%). A transitional pattern was characterized by any nonring-like or nondot-like cytoplasmic staining pattern. Subtypes were categorized as follows: DG, defined as a perinuclear pattern ≥ 70% or a dot pattern ≤ 8%; SG-A, defined as a dot pattern ≥ 70% (15).

### 2.5 Data analysis and statistical methods

The statistical analysis was performed via IBM SPSS statistics subscription software version 29 (International Business Machines Corp., Armonk, NY, USA).

For distribution, quantitative variables are presented as either the mean ± standard deviation (SD) or the median with the interquartile range (IQR). Data distribution was evaluated via the Shapiro-Wilk test, and quantitative variables were evaluated via Student’s *t* test or the Mann-Whitney *U* test. For qualitative variables, we used the *X^2^* test or Fisher’s exact test. The frequencies of the categorical variables (sex, histological type, and adenoma size—macro- or microadenomas) are presented as percentages. Spearman’s coefficient was used to verify correlations between numerical variables. Survival curves were plotted via Kaplan-Meier analysis considering the time of diagnosis and the date of the last follow-up. Disease-free survival was compared between the groups with Ki-67>3% or Ki-67<3% and the four lineage subtypes of tumors by the log-rank test. Cox proportional hazard analysis was used for multivariate analysis. For multivariable Cox regression analysis, the proportional hazards assumption was checked and verified via a global goodness-of-fit test proposed by Schoenfeld. The level of significance adopted for the statistical tests was 5% (p < 0.05).

Model selection for long-term survival was performed via a Cox proportional hazards model with forward selection considering age, sex, CSI, Knosp grade, tumor diameter, PitNET subtype, Ki-67 index > 3%, SSTR expression, and postoperative hormonal values (IGF-1, GH and PRL). Youden index calculations were used to determine the predictive cutoffs.

## 3 Results

### 3.1 Patient characteristics

The clinical, radiological and biochemical characteristics of the patients are presented in **Table 2**. A total of 50 patients were included, 54.4% of whom were women. On the basis of the biochemical evaluation, the majority of the patients had GH hypersecretion (67.3%), 8 (14.5%) had mixed PRL and GH hypersecretion, and 5 patients (9.1%) had only PRL hypersecretion. The majority of the tumors were macroadenomas (72%), with a median maximal tumor diameter at diagnosis of 22.85 mm. More than half (54.5%) presented suprasellar extension and cavernous sinus invasion (CSI). In terms of the modified Knosp grade, 25.5% of the samples were Grade 2, 16.4% and 7.3% were Grade 3, and Grade 4.

**Table 2:** Clinical, radiological and biochemical characteristics of the subjects included in the study.

	Distribution, n (%)
Age at diagnosis (years)*	45.52 ± 12.64
Women, n (%)	30 (54.5%)
Men, n (%)	20 (36.4%)
Biochemical diagnosis	
PRL- hypersecretion	5 (9.1%)
GH- hypersecretion	37 (67.3%)
GH- & PRL- hypersecretion	8 (14.5%)
PRL level at diagnosis, (ng/dl)	5987 (7.15, 111,.1)
IGF1 level at diagnosis, (xULN)	3.41 (2.29, 4.29)
GH level at diagnosis (ng/ml)	15.30 (3.87, 20.67)
Preoperative characteristics	
Tumor dimensions	
Maximal tumor diameter at diagnosis (mm)	22.85 (14, 29)
Microadenoma, n (%)	9 (18%)
Macroadenoma, n (%)	36 72%)
Giant adenomas, n (%)	5 (10%)
Knosp Grade	
0, n (%)	12 (21.8%)
1, n (%)	10 (18.2%)
2, n (%)	14 (25.5%)
3, n (%)	9 (16.4%)
4, n (%)	4 (7.3%)
Suprasellar extension	30 (54.5%)
CS invasion	20 (36.4%)
SCO (preoperative)	16 (29.1%)
Preoperative pituitary insufficiency, n (%)	17 (30.9%)
Apoplexy, n (%)	2 (3.6%)
Presurgical treatment	
Medication	8 (14.5%)
SSA	4 (12.5%)
DA	
Radiotherapy	
Surgical intervention	
Approach	45 (81.8%)
TS	5 (9.1%)
2 X TS	
Radiotherapy	
GK	6 (10.9%)
CRT	2 (3.6%)
Postoperative characteristics	
Follow-up	
Follow-up from diagnosis (years)*	7.02 (3, 10)
Surgical cure, n (%)	4 (12.5%)
Radiotherapy, n (%)	4 (12.5%)
Tumor progression after surgery, n (%)	5 (9.1%)
Postoperative complications, n (%)	7 (12.6%)
Transient AVP deficiency	4 (7.2%)
Epistaxis	1 (1.8%)
CSF fistula	2 (3.6%)
Postoperative pituitary insufficiency, n (%)	4 (7.2%)
Postoperative PRL level, (ng/dl)	265.90 (5.45, 16.83)
Postoperative IGF1 level (x ULN)	2.22 (1.37, 2.57)
Postoperative GH level (ng/ml)	5.06 (0.51, 3.82)
Postoperative maximal tumor diameter (mm)	13.46 (7.75, 18.5)
Postsurgical medical treatment, n (%)	34 (61.8%)
Postsurgical radiotherapy, n (%)	4 (12.5%)
Control of disease under medical treatment, n (%)	40 (80%)
Resistant to medical treatment, n (%)	6 (10.9%)

GH, growth hormone; PRL, prolactin; ACTH, adrenocorticotropic hormone; TSH, thyroid-stimulating hormone; FSH, follicle-stimulating hormone; LH, luteinizing hormone; Pit-1, pituitary-specific transcription factor PIT-1; CS, cavernous sinus; SCO, optic chiasm syndrome; SSA, somatostatin analogs; DA, dopamine agonists; TS, transsphenoidal; GK, gamma knife radiosurgery; CRT, three-dimensional conformal radiation therapy; AVP, antiduretic hormone; CSF, cerebrospinal fluid; ULN, upper normal limit.

*For continuous variables, values are presented as the median ± IQR (interquartile range).

Sex-related differences were observed in the study population: males had larger tumors (mean maximal tumor diameter at diagnosis: 26.11 mm for males versus 20.6 mm for females, p=0.040). The diagnostic value of IGF-1 was also greater in males (p=0.061). Among all patients, the 4 who were cured after neurosurgical intervention were females (p=0.346). Postoperative evaluation did not reveal a sex-related difference in biochemical remission. The results of the comparison between female and male patients are presented in **Table 3**.

**Table 3:** Comparative analysis of female and male patients.

	Females	Males	P
Age at diagnosis (years)	47.6 (39, 58)	42.40 (32.25, 51.5)	0.127
Maximal tumor diameter at diagnosis (mm)	20.6 (13, 26.25)	26.11 (17.45, 32.25)	0.040
PRL at diagnosis (ng/ml)	370.54 (8.005, 107)	13377 (7.04, 186.21)	0.596
IGF-1 at diagnosis (x ULN)	3.18 (2.33, 4.74)	3.77 (3.72, 5.61)	0.061
GH at diagnosis (ng/ml)	14.21 (3.88, 31.7)	17.40 (7.49, 16.47)	0.847
IGF-1 postoperative (x ULN)	2.06 (1.28, 2.08)	2.53 (1.84, 2.68)	0.147
GH postoperative (ng/ml)	4.82 (0.38, 3.90)	5.53 (0.92, 3.00)	0,647
PRL postoperative (ng/ml)	332.4 (5.63, 27.72)	160.22 (5.46, 107)	0.904
Maximal tumor diameter (postoperative)	12.66 (7, 18)	15.16 (11, 20.55)	0.147
Duration of medical therapy (years)	3.32	5.16	0.089
Ki-67	3 ± 0.89	2.72 ± 0.95	0.280

GH, growth hormone; PRL, prolactin; ULN, upper normal limit.

### 3.2 Histopathological and IHC evaluation

**Figure 1:**
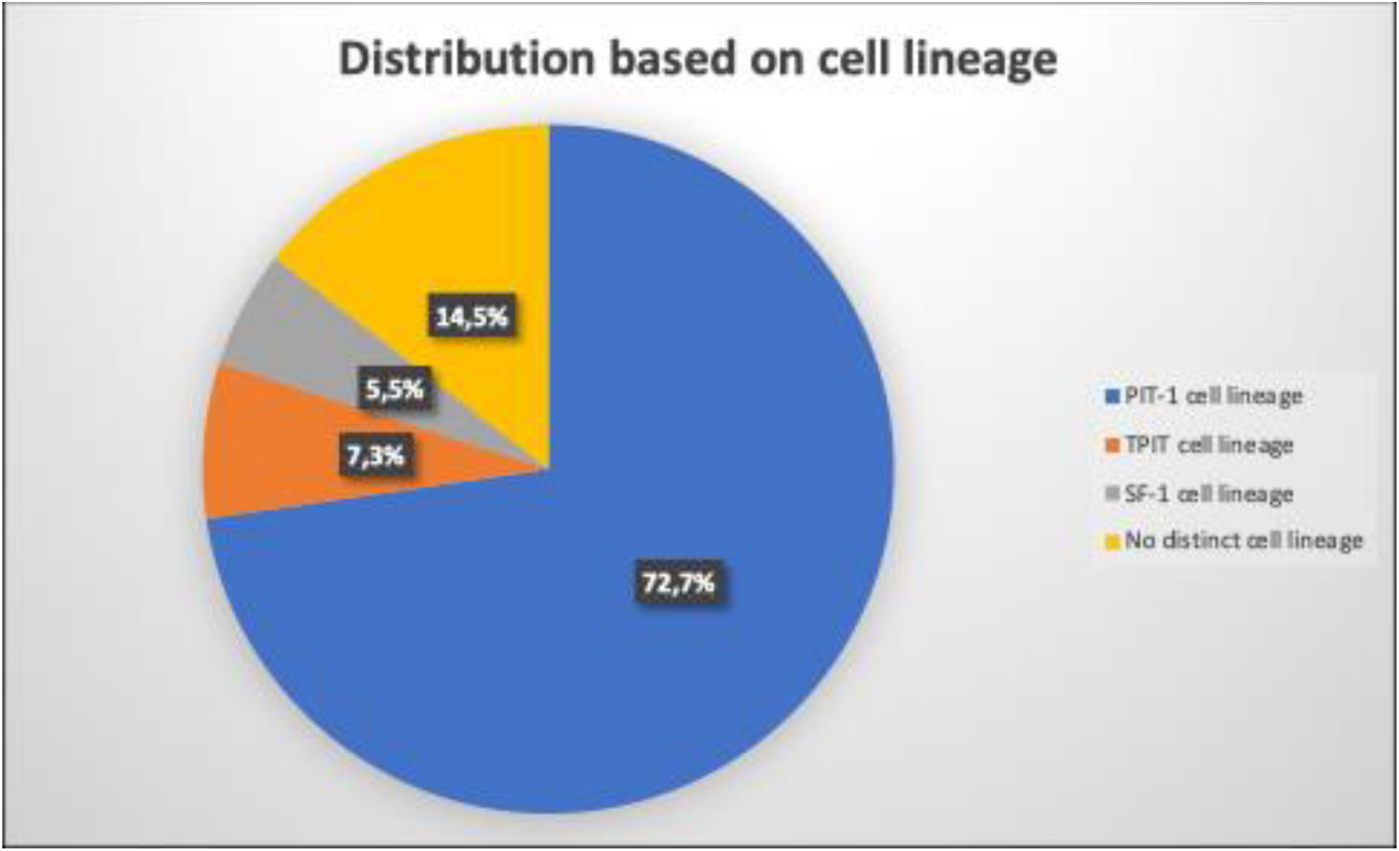
Distribution of PitNETs according to the WHO 2022 Classification.

**Figure 2:**
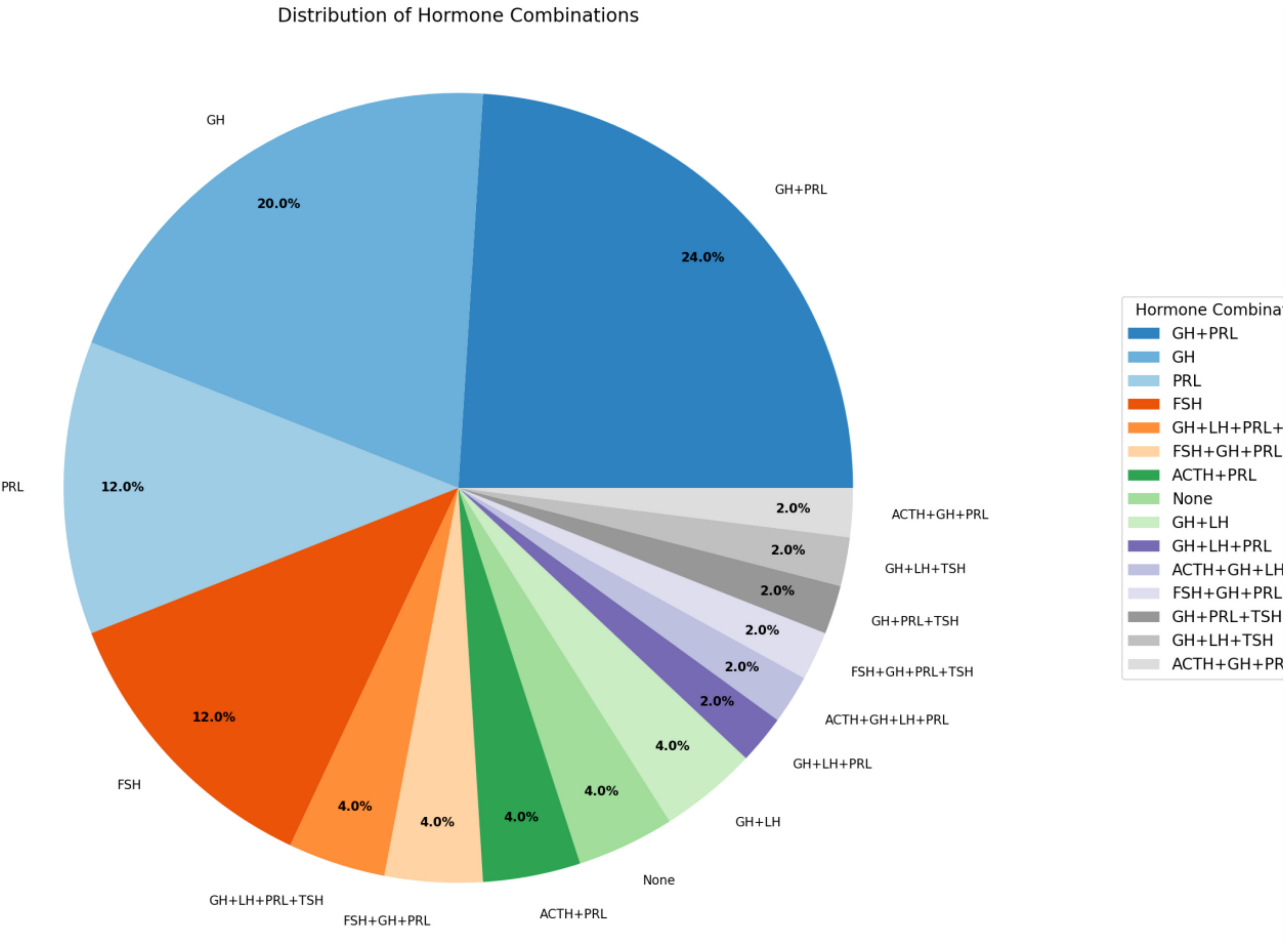
Distribution of IHC hormone combinations.

H&E staining revealed that most samples were acidic, with 11 mixed samples (acidophils and chromophobe) and 7 chromophobe samples. The main architectural pattern was diffuse/solid (**Figure 1**, **Table 4**). We followed the WHO classification to group the somatotroph tumors according to their IHC characteristics (**Table 5**). IHC staining for transcription factors (TFs) revealed that 40 patients were positive for PIT-1, 4 for TPIT and 3 for SF-1. Four patients were negative for all the TFs (**Figure 2**). Additionally, dual staining was found in 4 cases for PIT-1 and TPIT and 3 cases for PIT-1 and SF-1. In these patients, IHC for anterior pituitary hormones correlated with the first combination of TF in only one patient (**Table 6**). All tumors with dual staining for TF were densely granulated (DG) and had a ki-67 labeling index <3%.

**Table 4:** H&E* staining.

Categories	Distribution
Tinctoriality, n (%)	
Acidophil	32 (52.93%)
Cromophobe	7 (12.7%)
Mixed (acidophil and cromophobe)	11 (34.37%)
Pattern, n (%)	
Pseudoglandular (acinar)	2 (3.6%)
Papillary	12 (21.8%)
Trabecular	2 (3.6%)

**Table 5:** IHC* Classification.

Adenoma type	CAM 5.2 expression pattern	Pituitary hormones	Number (%)
Somatotroph adenomas	Densely granulated	GH	3 (6%)
	Sparsely granulated	GH	3 (6%)
	Mixed pattern	GH	3 (6%)
Mammosomatotroph adenomas	Densely granulated	GH + PRL	5 (10%)
	Sparsely granulated	GH + PRL	6 (12%)
	Mixed pattern	GH + PRL	3 (6%)
PRL- secreting adenomas	Densely granulated	PRL	2 (4%)

**Table 6:** Dual-transcription factor-stained pituitary adenomas in the study population.

	PIT-1 & TPIT	PIT-1 & SF-1
No cases	4	3
ACTH (+)	1	0
PRL (+)	2	3
GH (+)	4	3
FSH (+)	1	0
LH (+)	1	0
TSH (+)	1	1
CAM 5.2 pattern	DG	DG
Ki-67>3%	0	0

**Figure 3:**
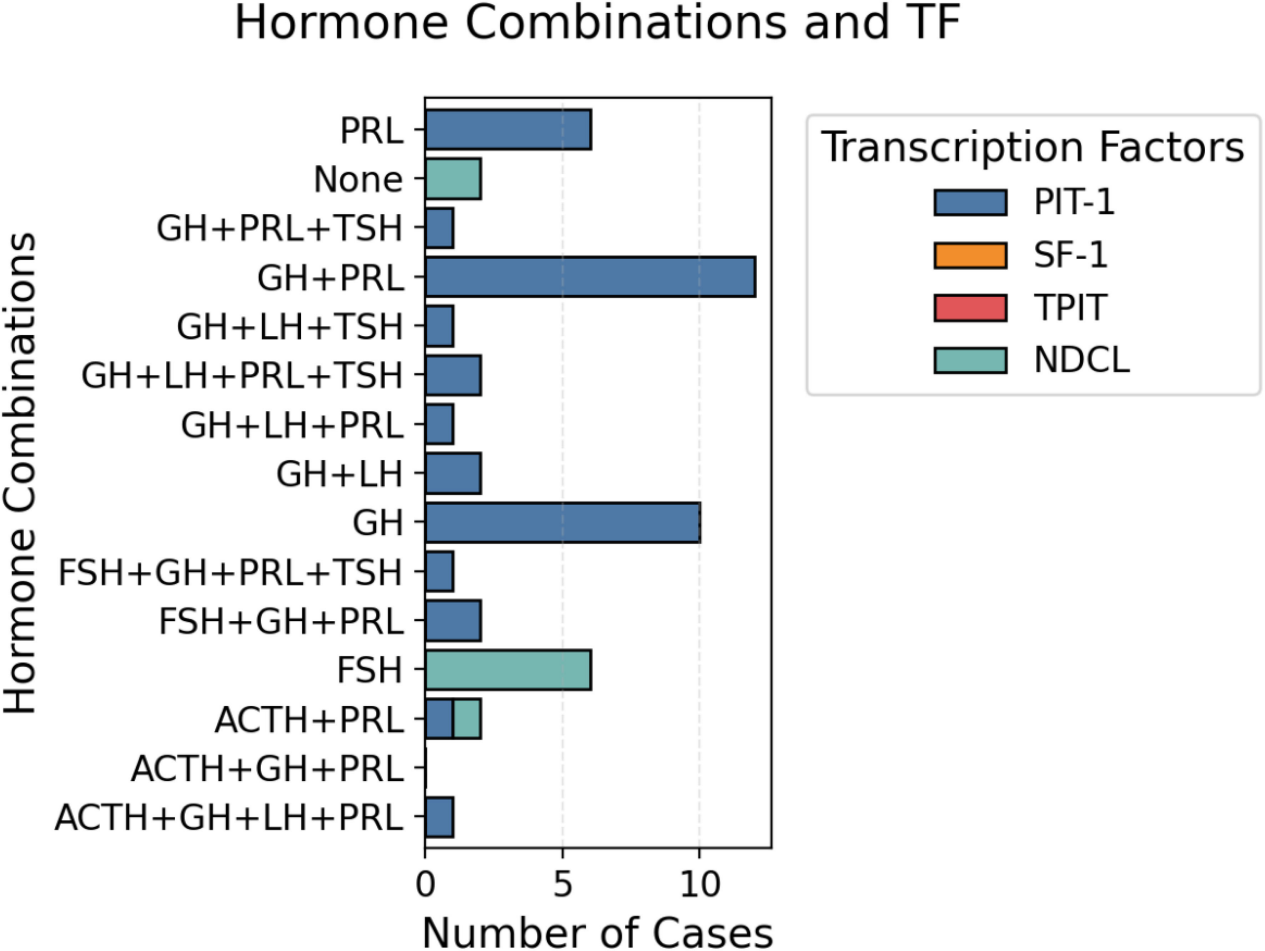
Distribution of hormone combinations and TFs. Representative images of a patient with a PIT-1 lineage tumor and intense positive GH staining.

Anterior pituitary hormone staining was positive as follows: PRL in 29 patients (52.6%) with a medium-intensity score of 1.06 ± 1.11, GH in 34 patients (61.8%) and strong-intensity staining in 52.7% of patients, TSH in 5 patients (9.1%), medium-intensity score of 0.20 ± 0.63, ACTH in 4 patients (7.3%), medium-intensity score of 0.08 ± 0.27, FSH in 3 patients (5.4%), medium-intensity score of 0.09 ± 0.36 and LH in 7 patients (12.7%) with a medium-intensity score of 0.26 ± 0.36. The other hormone combinations used in our study were as follows: GH+PRL, GH+PRL+TSH, GH+LH, ACTH+PRL, and ACTH+GH+LH+PRL. **Figure 3** shows the distribution of the hormonal combinations. Twenty percent of the patients were positive for only GH. The correlation between anterior pituitary hormones and TF was poor: 2 cases were null cell tumors (only hormonal staining was used), and after staining for TF, we identified 4 cases of tumors with no distinct lineage (**Figure 3**).

**Figure 4:**
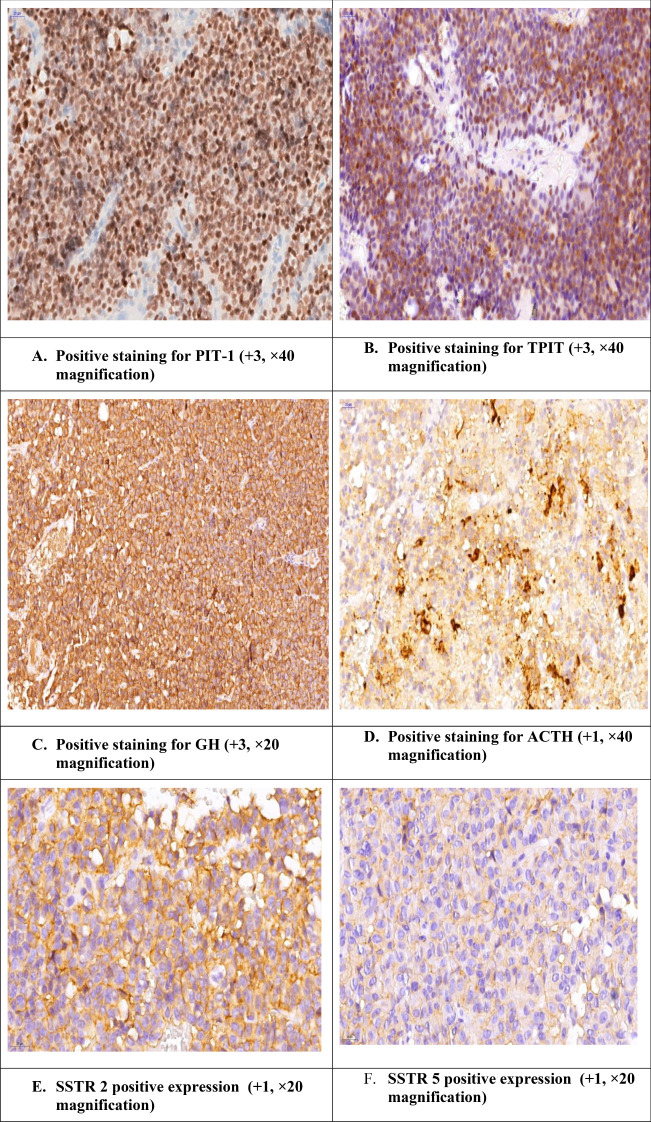
Acromegaly patient with positive staining for PIT-1 and TPIT and positive ACTH. The case of a female patient diagnosed with a GH-secreting PitNET who underwent transsphenoidal intervention and after surgery developed Cushing’s features. Additionally, during the endocrinological work-up, we observed a lack of decrease in the cortisol value after the test with 2x2 mg of dexamethasone.

The clinicopathological and pathological characteristics of the patients were compared according to PitNET subtype (**Table 7**). Significant differences were found among the 4 groups in terms of the maximal tumor diameter measured at the postoperative follow-up (p<0.01) and in terms of a ki-67 labeling index >3% (p=0.028). We analyzed the cure status among the tumor subtypes; 2 patients with PIT-1 lineage tumors were cured, and the other 2 had no distinct cell lineage identified (NDCL). Gross total resection (GTR) was achieved in 4 PIT-1 lineage tumors and 2 tumors with NDCL. Among the PIT-1-positive patients, 19 were SSTR5 positive, and 31 were SSTR2 positive. On the basis of CAM 5.2 expression, 10 tumors were DG, 8 were SG, and 6 had a mixed pattern: DG + SG. Among the TPIT-positive tumors, 2 were SSTR5 positive, 3 were SSTR2 positive, and all the tumors presented a DG pattern. Among the SF-1 tumors, 1 patient was SSTR5 positive, 3 patients were SSTR2 positive, and all were DGs (**Figure 4**).

**Table 7:** PitNET subtype and clinicoradiological characteristics.

	PIT-1 lineage (n=40)	TPIT lineage (n=4)	SF-1 lineage (n=3)	No distinct cell lineage (n=4)	P value
Age at diagnosis (years)	44.64 (34.5, 54.75)	41,67 (35, 60.25)	42.33 (35, 60.25)	44.63 (41, 61.5)	0.678
Maximal Tumor Diameter (mm) at diagnosis	23.76 (14.5, 30)	22.83 (6.62, 30)	17.90 (16.8, 30)	20.33 (9.65, 25)	0.481
Maximal Tumor Diameter (mm) Postoperative	13.91 (8.15, 18.75)	12.85 (5.8, 22.25)	10.93 (0, 10.93)	22.12 (3.75, 23.5)	<0.01
CSI	17	2	1	3	0.665
SCO (persistent)	8	1	0	0	0.416
Suprasellar extension	23	1	1	4	0.505
Ki-67>3%	9	0	0	0	0.028
GTR	4	0	0	2	1
Cured	2	0	0	2	1

CSI, cavernous sinus invasion; SCO, optic chiasm syndrome; GTR, gross total resection.

Somatostatin receptor 2 and 5 (SSTR2, SSTR5) expression was also evaluated. SSTR5 was positive in 20 patients (45.5%), with a medium-intensity score of 0.82 ± 1.13, and SSTR2 was positive in 34 patients (66.7%), with a medium-intensity score of 3.92 ± 4.45. Among all the patients, 14 had positive expression for both receptors.

CAM 5. 2 expression evaluations revealed that the DG pattern was present in 10 patients (18.2%), the SG pattern in 9 patients (16.4%), the mixed pattern (DG + SG) in 6 patients (10.9%), and the CAM 5.2 pattern was negative in 17 patients (30.9%). Among all the cases analyzed, 4 (7.3%) cases were considered controls (normal pituitary tissue and no tumoral tissue were present on the paraffin blocks). The Ki-67 labeling index had a medium intensity score of 2.89 ± 0.92. We compared the tumors with ki-67>3% and those with ki-67<3%, and significant differences were observed regarding the maximal tumor diameter measured at diagnosis (the tumors with a higher ki-67 had larger dimensions, p=0.048), but no differences were observed regarding the postoperative residual tumor (p=0.275). Additionally, differences were observed regarding the PRL level at diagnosis (p=0.038), and tumors with Ki-67>3% had higher values.

Correlations were calculated via Spearman’s rank correlation coefficients, significant correlations were found between age at diagnosis and ki-67 (rho=0.372, p<0.001), maximal tumor diameter at diagnosis and ki-67, PRL value at diagnosis, IGF-1 and GH at diagnosis (p<0.001) and postoperative tumor diameter. Additionally, biochemical remission (postoperative hormonal values) correlates with ki-67, tumor diameter and hormonal values at diagnosis.

For categorical variables, the significant chi-square test results indicate that preoperative treatment with dopamine (DA) agonists and control of the disease under medical treatment are significantly associated with a cured outcome (p<0.05). These results suggest that these variables may influence patient evolution.

**Figure 5:**
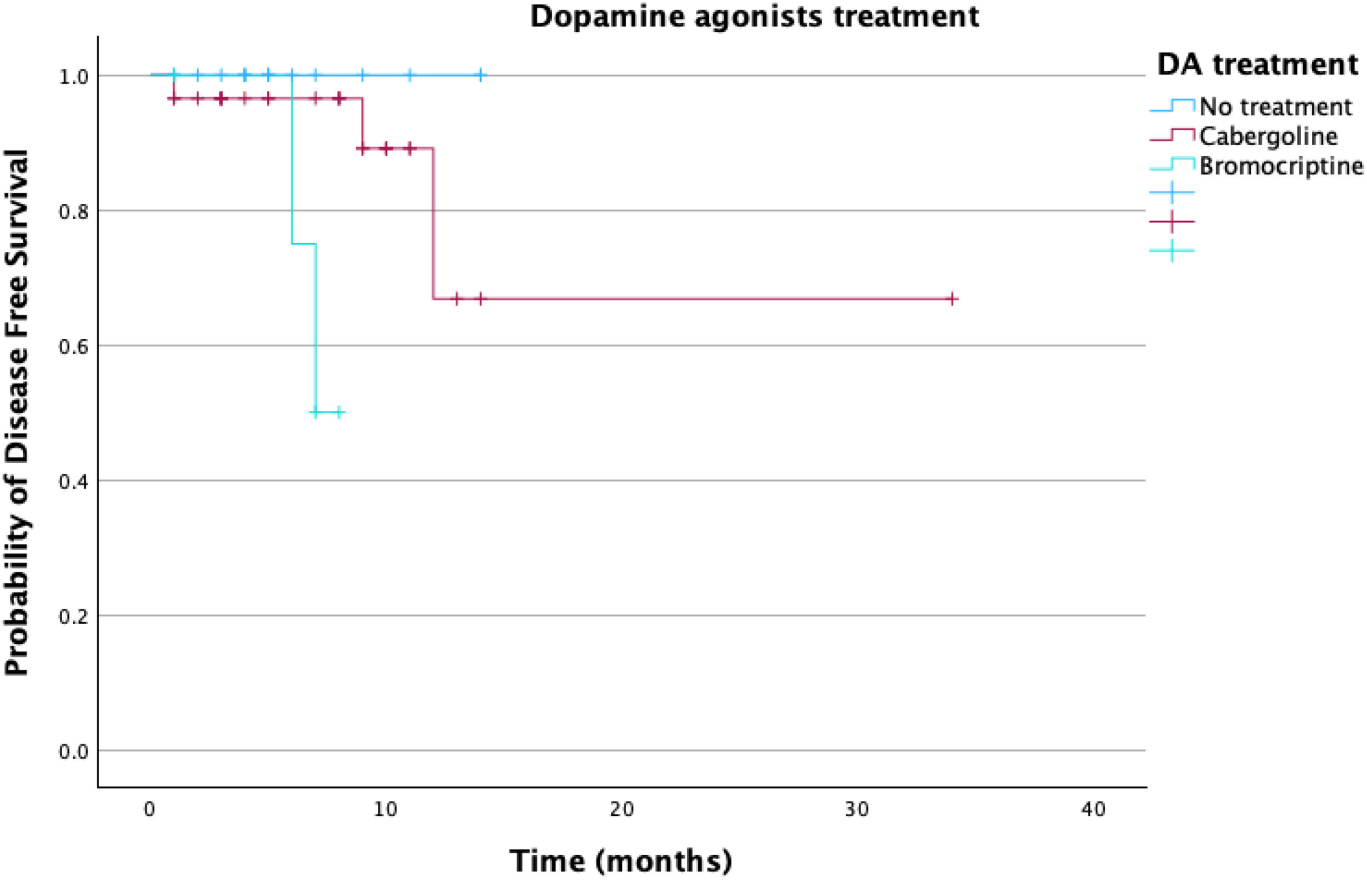
Kaplan-Meier survival analysis1. DA – dopamine agonist; log rank p<0.01.

Over the median follow-up time, no significant differences in disease-free survival were observed between patients with positive expression for SSTR and those with negative expression (log rank p=0.928). Significant differences were observed for patients who underwent treatment with DA before and after neurosurgical intervention (log rank p=0.01) (**Figure 5**). No differences were observed regarding survival based on PitNET subtype or cell lineage.

In the multivariate Cox regression analysis, the postoperative GH value was independently associated with the outcome (HR 1.042, 95% CI 1.004–1.081, p=0.030), as were the postoperative PRL value (HR 1, 95% CI 1,1.001, p=0.019), the ki-67 level (HR 2.43, 95% CI 1.109–5.330, p=0.026), the postoperative tumor diameter (HR 1.038 95% CI 0.997–1.080, p=0.068) and the expression of SSTRs 2 and 5. In the multivariable analysis, only postoperative GH was significantly associated with the outcome.

On the basis of the correlations calculated previously, we used multivariate logistic regression analyses to determine which variables have prognostic value for patient outcome.

**Figure 6:**
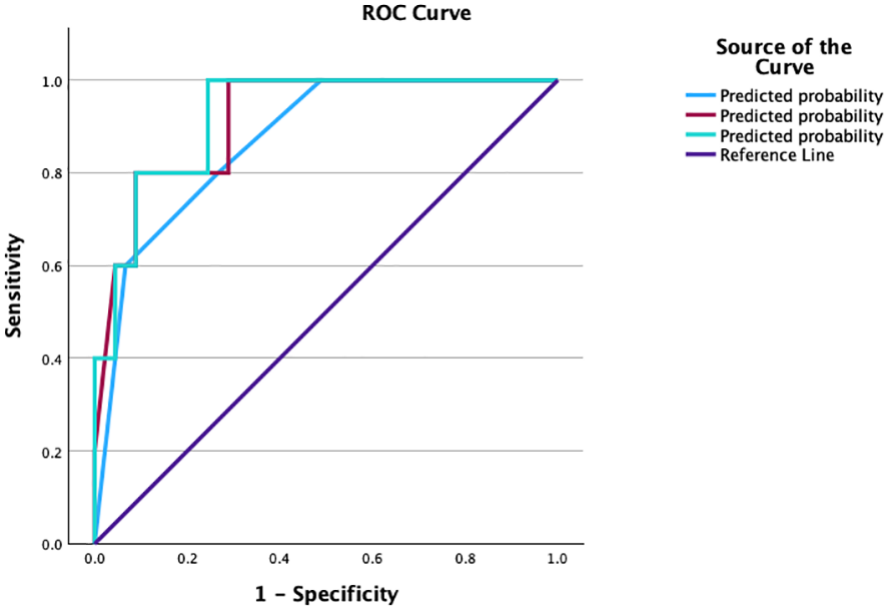
ROC curve compating the prognostic models evaluated. ROC, receiver operating characteristic curve; YI, Youden’s index; AUC, area under the curve.

The Ki-67 labeling index and SSTR 2 and SSTR 5 expression levels had good predictive performance, with area under the curve (AUC) values ranging from 0.5 to 0.867 (**Table 8**). Combining these three parameters, we established a model for predicting patient outcome with an AUC of 0.871 and relatively high sensitivity and specificity. When we added the postoperative values of GH and IGF-1 to the model, the prediction accuracy was greater, with an AUC of 0.916. The third model we tested included all the variables above plus the maximal postoperative tumor diameter and, in this case, had the highest predictive potential, with an AUC of 0.924 (**Figure 6**).

**Table 8:** Predictive value of parameters for patient outcome tested via ROC curves.

Variable	Sensitivity%	Specificity%	YI	AUC	Cutoff	p
Ki-67	80%	80%	0	0.5	> 0.8	1
SSTR 2	80%	31.1%	0.489	0.744	> 12.6	0.030
SSTR 5	80%	35.6%	0.444	0.722	> 11	0.052
GH postoperative	80%	11.1%	0.689	0.867	> 8.5	<0.05
IGF-1 postoperative (x%ULN)	80%	60%	0.200	0.724	> 6.7	0.041
Maximal Tumor Diameter postoperative	60%	17.8%	0.422	0.689	> 10.3	0.143

ROC, receiver operating characteristic curve; YI, Youden’s index; AUC, area under the curve; GH, growth hormone; IGF-1, insulin­like growth factor I; ULN, upper limit of normal.

A comparative analysis of the 3 models reveals a good prediction capacity (all p<0.05), but on the basis of the confidence intervals, the first model is slightly inferior, and the interval is greater than the other 2 (**Table 9**) (**Figure 5**).

**Table 9:** Comparison between the 2 prediction models.

	AUC	p	95% CI
Model 1	0.871	<0.05	0.73-1.011
Model 2	0.916	<0.05	0.808-1.023
Model 3	0.924	<0.05	0.830-1.019

## 4 Discussion

Predicting endocrine remission after neurosurgical treatment of patients with PR- and GH-secreting PITNETs is essential for decision making and choosing the best treatment pathways: surgery, medication and radiotherapy. Currently, we do not have a consensus regarding the prognostic markers that should be mandatorily evaluated.

In our study, we evaluated 50 patients diagnosed with GH- and PRL-secreting PitNETs. Using clinicopathological characteristics, we established 3 prognostic models that can predict patient evolution. Our results combined the Ki-67 labeling index, SSTR 2 and SSTR 5 expression, postoperative values of GH and IGF-1 and postoperative maximum tumor diameter.

We used the 2022 WHO classification for the cases included in our study. TF evaluation is essential for the classification of PitNETs, using this classification, and also clinical and imaging data showed important prognostic correlations. The clinical significance of the TF evaluation was disputed by some experts, but our study demonstrated that the IHC evaluation of TF and pituitary hormones alongside with the Knosp classification, ki-67 labelling index, CAM 5.2 pattern and in the cases with PRL- secretion, the ER alpha expression can provide valuable insights regarding the evolution of the patients (16). The cases without a distinct lineage have a more aggressive behavior, fact proved also in our study (17, 18). The patients with NDCL tumors were invasive and were in less cases controlled under medical treatment.

The clinico-pathological classification (Trouillas et al) proposed in 2020 is the most used, but compared with the 2022 WHO classification, the criteria used to evaluate the proliferation are less available to clinicians. The histopathological and IHC examination has become a mandatory step in the management of PitNETs.

An interesting entity with controversies and incompletely understood mechanism represents the dual expression for two transcription factors (17). In many cases, there have been reported false positive staining for SF-1 (17, 18). The majority of studies report dual expression of SF-1 and PIT-1, followed by PIT-1 and TPIT (17, 19). Also, we do not know if we should consider these tumors plurihormonal. In some cases there is a expression of two TF without hormonal correspondence. TF and hormonal IHC expression is not enough to predict the aggressive behavior, so the WHO classification has a limitation in this circumstance. We tried to add other factors that can impact the evolution and to create a prognosis model that can be easy to use by clinicians. Plurihormonal tumors can have variable clinical and histological presentation (20). In our study, 4 cases had positive expression for PIT-1 and TPIT, but only one had positive ACTH expression, a patient who had resistance to medical therapy and aggressive behavior.

The granulation pattern subtype represents another well-studied prognostic factor. Granulation patterns are a factor correlated with a high recurrence rate of GH-secreting PitNETs. The histologic subtypes determined via low-molecular-weight keratin (CAM5.2) are not always accurate. In fact, many tumors contain a variable mixture of perinuclear, transitional, and dot (fibrous body) patterns. Data from the literature shows that SG occurs in younger patients (mean age 37-46 years) than DG does (mean 46-56 years), but other studies reported no difference (21–23). The sex difference is less consistent: some studies report an increased frequency in females in SGs (23). SG was also related to a reduced probability of IGF-1 normalization-augmented recurrence risk and a significant need for reintervention (24–28).

Experts tried to create prognostic models which were tested in many studies, most of them in a retrospective manner. A prediction model created on the basis of data collected from 501 patients revealed that the CSI, ki-67 index and tumor diameter represent the most important factors that should be included in a prediction model. Two prognostic models were identified: M1 (tumor diameter ≥ 2.9 cm, CSI, and ki-67 > 3%), which is suitable for macroadenomas, and M2 (CSI and ki-67 > 3%), which is better at identifying at-risk smaller tumors (29). Machine learning methods and artificial intelligence are new tools that can be used for prediction.

Ki-67 has been associated with persistent disease or recurrence in many studies, but the cutoff is the subject of debate. The 3% cutoff was initially proposed by Thapar et al. (30–32). The correlation of ki-67 with tumor invasiveness has been supported by the WHO. Other studies reported lower Ki-67 cutoff values (1.0–2.0%) (33, 34). High ki-67 levels are independently related to long-term tumor outcomes.

Biochemical remission (postoperative hormonal values) correlated with ki-67, tumor diameter and hormonal values at diagnosis. In the literature, robust data suggest that a larger maximum tumor diameter is associated with a lower chance of early biochemical remission. A larger maximum tumor diameter and higher random GH concentration at diagnosis are associated with a lower chance of long-term remission (25, 35–37). In the cases of PRL secreting PitNETs, surgery and female gender were independent predictors of control of hyperprolactinemia (38). In women symptoms such as amenorrhea are investigated at an early time-point, and prolactin levels are usually not as high as in men (35, 39–42).

There is no algorithm that can give all solutions in predicting early and long-term outcome in patients diagnosed with PitNETs with first-line surgery.

ER∝ expression is another prognostic factor that has been less explored. It can be used to predict the prognosis for pure PRL- secreting PitNETs or for those with mixed GH- and PRL- expression. It has been correlated with postoperative PRL levels and had lower intensity in female patients (41).

The main limitation of our study is its retrospective nature. We were also limited by the use of different neurosurgical techniques and the availability of the tumor tissue found on the paraffin-embedded blocks between centers. In addition, our study lacked data on radiological parameters such as T2-signal intensity and p53 expression. However, these limitations are common in medical research and reflect the nature of daily practice. A major strength of our study is that this is one of the few analyses from our country that corroborates the clinical and biochemical data with histopathological and IHC evaluations. Additionally, this is the first prediction model that was developed using a cohort from our country. Our study highlights that combining a diverse set of classification algorithms to predict the outcome of first-line surgery in patients diagnosed with GH- and PRL- secreting PitNETs.

## 5 Conclusions

Tumor size, tumor invasion, the ki-67 labeling index, the expression of the SSTR and postoperative evaluation (hormonal work-up and imaging studies) are the most important factors that can predict long-term postoperative evolution. Additionally, the TF-based classification of PitNETs remains an important step in the prognosis-based approach for these tumors. Certain histologic subtypes remain predictors, but the risk of progression may not be identified only by this approach. A multistep evaluation should be recommended in these cases.

## Data Availability

The original contributions presented in the study are included in the article/supplementary material. Further inquiries can be directed to the corresponding author.
